# A sword or a buffet: plant endomembrane system in viral infections

**DOI:** 10.3389/fpls.2023.1226498

**Published:** 2023-08-11

**Authors:** Ivana Jovanović, Nicole Frantová, Jan Zouhar

**Affiliations:** ^1^ Department of Crop Science, Breeding and Plant Medicine, Faculty of AgriSciences, Mendel University in Brno, Brno, Czechia; ^2^ Central European Institute of Technology, Faculty of AgriSciences, Mendel University in Brno, Brno, Czechia

**Keywords:** plant virus, endomembrane system, endoplasmic reticulum, virus replication, autophagy

## Abstract

The plant endomembrane system is an elaborate collection of membrane-bound compartments that perform distinct tasks in plant growth and development, and in responses to abiotic and biotic stresses. Most plant viruses are positive-strand RNA viruses that remodel the host endomembrane system to establish intricate replication compartments. Their fundamental role is to create optimal conditions for viral replication, and to protect replication complexes and the cell-to-cell movement machinery from host defenses. In addition to the intracellular antiviral defense, represented mainly by RNA interference and effector-triggered immunity, recent findings indicate that plant antiviral immunity also includes membrane-localized receptor-like kinases that detect viral molecular patterns and trigger immune responses, which are similar to those observed for bacterial and fungal pathogens. Another recently identified part of plant antiviral defenses is executed by selective autophagy that mediates a specific degradation of viral proteins, resulting in an infection arrest. In a perpetual tug-of-war, certain host autophagy components may be exploited by viral proteins to support or protect an effective viral replication. In this review, we present recent advances in the understanding of the molecular interplay between viral components and plant endomembrane-associated pathways.

## 1 Introduction

Plant cells contain a sophisticated system of membrane-limited subcellular compartments that guarantee a spatial-temporal separation of their specific functions and chemical compositions, called the endomembrane system. The endoplasmic reticulum (ER) is one of the largest structures in eukaryotic cells, comprising interconnected tubules, cisternae, and three-way junctions (Sparkes et al., 2009). The constricted tubules pass through plasmodesmata, making endoplasmic reticulum a *bona fide* multicellular organelle (Nicolas et al., 2017). Regarding its function, endoplasmic reticulum is a gateway involved in lipid metabolism, and synthesis and folding of proteins. Each plant cell may contain hundreds of individual Golgi stacks that actively move along the cytoskeleton (Boevink et al., 1998). The cellular functions of plant Golgi include synthesis and trafficking of complex carbohydrates for the cell wall, and participation in transport and modifications of proteins destined for other organelles, such as vacuoles and chloroplasts (Villarejo et al., 2005; Oikawa et al., 2013; Di Sansebastiano et al., 2017). The *trans*-Golgi network (TGN) is a major sorting station in plant cells, governing cargo delivery to the plasma membrane and participating in post-Golgi trafficking to endosomes and the vacuole (Ruiz Rosquete et al., 2018). In addition, plant TGN also functions as an early endosome, receiving endocytosed cargo from the plasma membrane (Viotti et al., 2010). The exact contribution of various post-Golgi compartments and their resident proteins to protein sorting constitutes still an unresolved area of plant cell biology. To conciliate the contradictory experimental data, a unified model was recently proposed (Zouhar et al., 2023). It states that a distinct population of TGN, termed late TGN, is converted into early multivesicular bodies (MVBs). These early MVBs then gradually mature into late MVBs that finally fuse with the tonoplast, delivering its content into the vacuolar lumen. Vacuole represents a dominant structure in plant cells that can occupy up to 90% of the cell volume (Zhang et al., 2014). It is a multifunctional organelle and in vegetative cells, its primary lytic function resembles that of yeast vacuoles and lysosomes in metazoans. However, certain cell types may contain protein storage vacuoles (PSVs) with neutral pH, structures that are unique to plants (Frigerio et al., 2008). Plants as sessile organisms mastered the use of their endomembrane system pathways to respond to various environmental stresses. Experimental data indicate a critical role of the plant endoplasmic reticulum (Park and Park, 2019), the *trans*-Golgi network (Ruiz Rosquete and Drakakaki, 2018), the autophagosomes and multivesicular bodies (Wang et al., 2020) in responses to both abiotic and biotic stresses.

Plant RNA and DNA viruses represent half of the disease-causing pathogens with a tremendous economic impact on agriculture (Nicaise, 2014). Positive-sense single-strand (+ss) RNA viruses are among the most economically damaging pathogens. Within this group, viruses of the *Closterovirus*, *Cucumovirus*, *Luteovirus*, *Potyvirus* and *Sobemovirus* genera cause serious diseases in various crops, including crucial cereals, vegetables but also in high-value fruit crops, such as citrus and papaya (Tatineni and Hein, 2023). Upon infection, RNA viruses induce formation of specialized cytoplasmic viral replication organelles (VROs) to facilitate their replication, to achieve cell-to-cell movement and to shelter their replication complexes from host antiviral defense. The endoplasmic reticulum is a primary target for plant RNA viruses, but they frequently remodel additional cellular compartments, such as chloroplasts and peroxisomes (Richardson, 2019). Begomoviruses constitute a remarkably successful group of emerging viruses and carry a monopartite or bipartite single-strand (ss) DNA genome (Fiallo-Olivé and Navas-Castillo, 2023). Their replication does not require a drastic host endomembrane remodeling and takes place in the nucleus. Eleven species within the *Cassava mosaic virus* (genus *Begomovirus*) group infect cassava, a daily staple in the diet of more than half a billion people in Africa and India (Legg et al., 2015).

To control virus infections, plants deploy two major strategies. The highly conserved primary antiviral response is the RNA interference (RNAi), initiated by plant DICER-LIKE enzymes (DCLs) that cleave long double-strand RNAs or RNA hairpins into small interfering RNAs (siRNAs) of distinct lengths. The siRNAs associate with ARGONAUTE proteins, catalytic components of the silencing complexes, which mediate methylation of viral DNAs, silencing of viral RNAs, or an inhibition of the viral RNA translation (Yang and Li, 2018). The crucial role of RNAi is emphasized by the fact that as a counter-acting measure, viruses evolved silencing suppressors that act at various steps of antiviral silencing but may also play other roles during infection (Csorba et al., 2015). The second major antiviral strategy is represented by effector-triggered immunity (ETI). During ETI-based antiviral response, host resistance proteins (R) recognize viral avirulence (Avr) factors, resulting in production of reactive oxygen species (ROS), induction of salicylic acid and expression of defense-related genes (Huang, 2021). Recent discoveries of a selective viral protein degradation by autophagy and the ubiquitin-proteasome pathway, and the reevaluation of the role of membrane-localized receptor-like kinases in antiviral defense exposed plant responses to viral infections as a complex multilayered system (Teixeira et al., 2019; Wu et al., 2019b; Dubiella and Serrano, 2021; Wu et al., 2021; Yang and Liu, 2022).

In this review, we summarize some of the important discoveries in the field of molecular plant virology related to plant endomembrane system. In the first section, we present examples of a misappropriation of various endomembrane compartments and their resident proteins by distinct viral genera to create viral replication organelles and an involvement of these novel membrane entities in the cell-to-cell viral movement. In the second part, we focus on membrane-localized receptors of viral molecular patterns, their putative localizations, and the identity of molecules they likely recognize. Finally, the third section introduces a dual role of plant selective autophagy during viral infections. Specific degradation of viral proteins often leads to an infection arrest, but plant viruses evolved mechanisms to utilize host autophagy processes to promote viral replication.

## 2 Hijacking the plant endomembrane system

Positive-strand RNA viruses perform their life cycle in the cytoplasm of an infected host cell, where they are met by the antiviral defense systems and unfavorable conditions for genome replication. Therefore, the virus must create an intracellular environment where viral and host proteins concentrate, facilitating its replication. In eukaryotic cells, such spatial and functional isolation is guaranteed in membrane-limited compartments. Indeed, RNA viruses induce biogenesis of such membranous replication entities, which display two basic topologies. The first type of viral replication organelles (VROs) is represented by membrane invaginations, also called spherules, while the second type is formed by membrane protrusions to generate tubules or vesicles (He et al., 2023). This is made possible by the ability of viral proteins to induce membrane deformations or to seize host proteins with similar capabilities (Agaoua et al., 2021). For optimal biogenesis and functioning of replication organelles, the RNA viruses coopt host proteins and modify or trigger host cellular pathways to fine tune the lipid and protein composition of these newly formed compartments (Ketter and Randall, 2019; He et al., 2023).

### 2.1 Manipulating host cellular pathways to support viral infection

Positive-strand RNA viruses often disrupt ER homeostasis as a first step in the formation of viral replication organelles that contain viral replication complexes (VRCs) and sites of viral particle assembly and maturation (Laliberté and Sanfaçon, 2010). One of the ER-associated pathways that is activated and utilized by plant viruses is the unfolded protein response (UPR) that represents a conserved eukaryotic reaction to a presence of misfolded proteins in the lumen of the endoplasmic reticulum. In Arabidopsis, the accumulation of misfolded proteins triggers INOSITOL REQUIRING ENZYME1 (IRE1)-mediated splicing of *bZIP60* mRNA that then encodes an active transcription factor (Nagashima et al., 2011). Similarly, upon detection of misfolded proteins, ER membrane-localized bZIP17 and bZIP28 proteins are transported to Golgi, where they are proteolytically processed to yield functional transcription factors that regulate expression of ER resident enzymes and chaperons to increase folding capacity in the ER (Che et al., 2010).

*Potato virus X* (PVX, +ssRNA, genus *Potexvirus*) infection triggers upregulation of many genes associated with the UPR, with the membrane-localized triple gene block protein 3 (TGBp3) identified as the responsible factor (Ye et al., 2011). Supporting a proposed proviral role of UPR in the PVX infection, silencing of *bZIP60* inhibits viral replication and delays virus accumulation in *Nicotiana benthamiana* (Ye et al., 2011). The 6K2 transmembrane protein of *Turnip mosaic virus* (TuMV, +ssRNA, genus *Potyvirus*) was also identified as a potent inducer of *bZIP60* splicing. Both *bzip60* and *ire1* mutants displayed alleviated viral pathogenesis, suggesting that IRE1-mediated *bZIP60* splicing is essential for the TuMV infection (Zhang et al., 2015). It was hypothesized that both TGBp3 and 6K2 trigger the UPR pathway because they might be considered chronically misfolded proteins by the host UPR sensors (Bao and Howell, 2017). Regarding the target genes upregulated by the UPR-generated transcription factors, tobacco plants infected with *Cucumber mosaic virus* (CMV, +ssRNA, genus *Cucumovirus*), *Soybean mosaic virus* (SMV, +ssRNA, genus *Potyvirus*), *Pepper mottle virus* (PepMoV, +ssRNA, genus *Potyvirus*) and *Potato virus X* (*Potexvirus*) demonstrated a significant up-regulation of genes encoding ER lumen-localized IMMUNOGLOBULIN BINDING PROTEIN 1 and 2 (BiP1 and BiP2), members of the heat shock protein 70 (HSP70) family. Tobacco BiP2 chaperon was identified as an interacting partner of potyviral nuclear inclusion protein b (NIb), which functions as the RNA-dependent RNA polymerase responsible for viral replication (Widyasari et al., 2023). Considering that NIb is a very active recruiter of various proviral host proteins (Shen et al., 2020), we may speculate that the observed UPR-mediated induction of BiP molecular chaperones has likely a direct role in folding of proteins essential for successful viral infection.

The virus-triggered UPR upregulation is not exclusive to viruses with +ssRNA genomes. The membrane-associated viral proteins NSvc2 and NSvc4 of *Rice stripe virus* (RSV, -ssRNA, genus *Tenuivirus*) activate the bZIP17/28 pathway in *N. benthamiana*. Silencing *bZIP17/28* delayed RSV infection and decreased virus accumulation, indicating that RSV triggers UPR to specifically promote its infection (Li et al., 2022). The βC1 protein of *Tomato yellow leaf curl China virus* (TYLCCNV, ssDNA, genus *Begomovirus*) is a significant inducer of UPR and can upregulate the *bZIP60* expression. Transient expression of bZIP60-regulated BiP and calreticulin molecular chaperones in *N. benthamiana* resulted in higher accumulation of viral DNA and more severe viral symptoms, suggesting that these chaperones positively assist the TYLCCNV infection (Zhang et al., 2023).

Biological membranes represent an essential platform for viral replication. While some viruses form their viral replication compartments from preexisting host membranes, others trigger general membrane proliferation and/or accumulation of specific lipids at the VROs (Verchot, 2011). Endoplasmic reticulum is a major cellular compartment responsible for lipid biosynthesis (Jacquemyn et al., 2017). As the formation of viral replication organelles requires a significant amount of new membranes, it is not surprising that ER membrane proliferation is a common phenotype observed during various plant virus infections (Schaad et al., 1997; Lee and Ahlquist, 2003; Turner et al., 2004). In plants, ER membrane proliferation is regulated by phosphatidate phosphohydrolase (PAH) (Craddock et al., 2015). The disruption of PAH leads to accumulation of phosphatidic acid (PA) and massive proliferation of ER membranes that can be efficiently utilized for the viral replication organelle formation (Zhang et al., 2018). It was shown that overexpression of Arabidopsis PAH in tobacco leads to decreased accumulation of *Tomato bushy stunt virus* (TBSV, +ssRNA, genus *Tombusvirus*), *Red clover necrotic mosaic virus* (RCNMV, +ssRNA, genus *Dianthovirus*), *Brome mosaic virus* (BMV, +ssRNA, genus *Bromovirus*) and *Tobacco mosaic virus* (TMV, +ssRNA, genus *Tobamovirus*) (Chuang et al., 2014; Zhang et al., 2018). However, no direct downregulation of PAH initiated by viral proteins has been reported yet. Interestingly, *Red clover necrotic mosaic virus* (*Dianthovirus*) induces a high accumulation of PA by recruiting host phospholipases D into viral replication complexes (Hyodo et al., 2015). Phosphatidic acid is not the only phospholipid that supports an efficient viral replication. When *Brome mosaic virus* (*Bromovirus*) infects yeast cells, the BMV 1a replication protein recruits Cho2p (choline requiring 2) enzyme to synthesize phosphatidylcholine (PC) at the site of viral replication (Zhang et al., 2016). Similar accumulation of PC at the viral replication sites was observed also for human poliovirus and hepatitis C virus, revealing a conserved attribute among +ssRNA viruses (Zhang et al., 2016). Considering the complexity of lipid metabolic pathways, plant viruses may constitute an invaluable tool in studying the lipid biosynthesis regulation in plants.

### 2.2 Establishing the viral replication organelles

For the formation of VROs, some plant RNA viruses specifically target particular membrane compartments, such as the ER, chloroplasts, and vacuoles. In contrast, other RNA viruses do not have these strict requirements and either target several membrane populations in the course of the infection or they appropriate other available membrane types as an alternative (Xu and Nagy, 2014).

*Brome mosaic virus* (*Bromovirus*) establishes its VROs through invaginations of the ER membrane. The formation of approx. 70 nm lumenal spherules requires the Endosomal Sorting Complex Required for Transport (ESCRT). The ESCRT machinery has membrane curvature-inducing properties and in later stage narrows the neck connecting the spherule with cytoplasm. BMV recruits the ESCRT proteins transiently through interaction with 1a replication protein (Diaz et al., 2015). *Beet black scorch virus* (BBSV, +ssRNA, genus *Betanecrovirus*) auxiliary replication protein p23 plays a crucial role in establishing ER-derived spherules in *N. benthamiana*. In a rare topology arrangement, these spherules are connected to each other by tubules (Cao et al., 2015).

Biogenesis of the *Potato virus X* (*Potexvirus*) VRO requires three partially overlapping open reading frames (ORFs), termed the triple gene block (TGB) (Morozov and Solovyev, 2003). In the PVX infected cell, VRCs localize to large perinuclear structures called X-bodies (Tilsner et al., 2012). The core of the X-body is formed by TGBp1 aggregates, surrounded by dense reticulated network of ER membranes recruited by the TGBp2/TGBp3 complex. The replication complexes are associated with the TGBp1 and TGBp2 aggregates, while the virions localize to the periphery of the X-bodies (Wu et al., 2019a).

*Barley stripe mosaic virus* (BSMV, +ssRNA, genus *Hordeivirus*) builds its VRO on chloroplasts and causes dramatic morphological changes of these organelles (Carroll, 1970). The BSMV replication complexes are associated with peripheral outer membrane-invaginated spherules and large cytoplasmic invaginations (CIs) (Carroll, 1970; Lin and Langenberg, 1985; Torrance et al., 2006). The peripheral spherules are often clustered within inner membrane-derived packets, and they are connected to the cytoplasm *via* neck-like structures (Jin et al., 2018). The CIs are associated with the later stage of BSMV infection. The transmission electron tomography revealed that the invagination of the chloroplast membranes progresses gradually in the course of the infection, resulting in the CIs of diverse morphologies (Jin et al., 2018).

The initial stage of the *Turnip mosaic virus* (*Potyvirus*) VRO biogenesis includes remodeling of the ER, mediated by 6K2 viral protein (Cotton et al., 2009). A detailed time-course analysis of the 6K2-driven biogenesis of the replication compartments revealed early accumulation of convoluted ER membranes, followed by a formation of single-membrane vesicle-like (SMVL) structures (Wan et al., 2015a). These highly motile compartments represent functional VROs that move on actin filaments through thin tubular structures that traverse the vacuole (Cotton et al., 2009; Grangeon et al., 2013). During the TuMV VRO biogenesis, the 6K2 transmembrane protein coopts the SEC24A subunit of the COPII coatomer at the ER exit sites (ERES), affecting the conventional ER-to-Golgi anterograde trafficking of the host cargo proteins (Wei and Wang, 2008; Jiang et al., 2015). Importantly, 6K2 also interacts with ROOT HAIR DEFECTIVE 3 (RHD3) at the endoplasmic reticulum membrane (Movahed et al., 2019b). RHD3 is a plant dynamin-like GTPase that is required for efficient homotypic fusion of ER membranes (Chen et al., 2011). Interaction of RHD3 with 6K2 is essential for the formation and maturation of the TuMV motile VROs, as mutations in the RHD3-interacting GxxxG domain of 6K2 result in the accumulation of 6K2 in the Golgi stacks, representing a nonproductive pathway for viral infection (Cabanillas et al., 2018; Movahed et al., 2019b). In agreement with the identified crucial role of RHD3 in the TuMV life cycle, the *rhd3* mutant plants exhibit compromised viral replication and cell-to-cell movement (Movahed et al., 2019b). The 6K2-induced motile VROs then target chloroplasts, multivesicular bodies (MVBs), vacuoles or plasma membrane to establish a secondary subset of VROs (Wei et al., 2010a; Wei et al., 2010b; Wan et al., 2015a; Cabanillas et al., 2018; Li et al., 2020a). At an early stage of infection, the 6K2 VROs are localized at the periphery of the chloroplasts, while later, TuMV-infected cells display tubular structures, consisting of approx. 20 chloroplasts with 6K2-containing VROs localized between adjacent chloroplasts (Wei et al., 2013). This unique localization may provide an ideal energy- and resource-rich environment for the TuMV replication. Formation of the tubular chloroplast aggregates depends on the SYP71 Qc-SNARE protein, which normally regulates membrane fusion processes at the ER and plasma membrane. However, as SYP71 is not essential for targeting of the 6K2 VROs to the chloroplast outer membrane, its molecular role in chloroplast clustering needs to be further elucidated (Wei et al., 2013). Chloroplasts are sites of salicylic and jasmonic acid biosynthesis and sources of ROS and calcium bursts, which makes them important organelles in antiviral responses (Littlejohn et al., 2021). In *N. benthamiana*, chloroplasts frequently migrate to perinuclear position during plant-pathogen interactions (Ding et al., 2019). This rearrangement likely facilitates an efficient regulation of expression of defense related genes *via* retrograde signaling (Chan et al., 2016). In a counteractive measure, the TuMV silencing suppressor protein VPg interacts with the chloroplast NADH dehydrogenase-like (NDH) complex M subunit (NdhM), resulting in a suppression of the antiviral chloroplast perinuclear clustering in *N. benthamiana* (Zhai et al., 2021). In Arabidopsis, targeting of the 6K2 VROs to the multivesicular bodies utilizes the VTI11 Qb-SNARE protein and VACUOLAR SORTING RECEPTOR4, bypassing the *trans*-Golgi network, a post-Golgi sorting organelle (Cabanillas et al., 2018; Wu et al., 2022a). In addition to the highly motile 6K2-labelled VROs and the secondary sites of viral replication, the TuMV infected cell contains a large perinuclear structure, which comprises amalgamated Golgi stacks, the endoplasmic reticulum, chloroplasts, COPII coatomers, and viral and host proteins necessary for replication (Grangeon et al., 2012). Elegant photoactivation experiments revealed that the perinuclear structure is both a target and a source of motile 6K2 VROs (Grangeon et al., 2012). The TuMV infection process is also accompanied by a formation of double-membrane tubules (DMTs), which were observed by transmission electron tomography. The DMTs function likely as the ultrastructure underlying the perinuclear compartment and the sites of the TuMV particle assembly (Wan et al., 2015a).

The key viral protein in the establishment of the *Tomato bushy stunt virus* (*Tombusvirus*) replication organelle is the p33 RNA chaperone, which interacts with approx. 100 host proteins (Nagy et al., 2012). The TBSV VROs are established by invaginations of the peroxisomal membranes (Fernández De Castro et al., 2017). This inward vesiculation is executed by the host ESCRT proteins recruited by p33 protein (Barajas et al., 2009). However, the tombusvirus replication requires an appropriation of numerous additional resources of the host cell (Nagy and Feng, 2021). Their efficient and rapid delivery to the VRO is ensured by p33 inhibition of cofilin, a major modulator of actin filament disassembly, resulting in the microfilament stabilization (Nawaz-Ul-Rehman et al., 2016). The p33-mediated establishment of ER-peroxisome membrane contact sites allows for generation of sterol-rich membrane environments, crucial for a successful TBSV replication (Barajas et al., 2014). TBSV also subverts ER-derived anterograde vesicles *via* p33 interaction with Rab1 GTPase, resulting in the accumulation of COPII vesicles at the replication compartments (Inaba et al., 2019). Interestingly, interactions of p33 with small GTPases extend beyond the Rab1 clade. The p33 protein interacts with the host Rab7 small GTPase, causing its relocalization into the VROs (Feng et al., 2021b). The change of Rab7 localization leads to mislocalization of the core retromer complex and mistargeting of multiple host lipid enzymes, including phosphatidylinositol 3-kinase, phosphatidylinositol 4-kinase and phosphatidylserine decarboxylase, into the replication organelle (Feng et al., 2021a). The enrichment of phosphatidylinositol-3-phosphate and phosphatidylinositol-4-phosphate in the VRO membrane is crucial for efficient TBSV replication (Feng et al., 2019; Sasvari et al., 2020). In addition, p33 mediates recruitment of Rab5-positive endosomes that leads to the enrichment of phosphatidylethanolamine in the VRO membranes (Xu and Nagy, 2016).

The tonoplast is appropriated for the VRO formation during infection of *Cucumber mosaic virus* (*Cucumovirus*). Confocal microscopy and electron tomography approaches revealed that CMV replication proteins 1a and 2a trigger formation of spherules 50–70 nm in diameter at the vacuolar membrane. The interior of these spherules communicates with cytosol through neck‐like channels (Wang et al., 2021). Recently identified interaction of 126kDa protein of *Tobacco mosaic virus* (*Tobamovirus*) with the SYP2 Qa-SNARE proteins and decreased accumulation of TMV in plants lacking the SYP2 syntaxins indicate that the vacuolar membrane plays an important role in the *Tobacco mosaic virus* life cycle (Ibrahim et al., 2020).

### 2.3 Cell-to-cell movement

For efficient intercellular movement, viral replication complexes of many +ssRNA viruses are targeted to plasmodesmata (PD). The colocalization of the viral replication machinery with viral movement proteins (MPs) to the PD greatly increases the chance of the MP interaction with virions or viral RNAs and decreases the host mRNA interaction with the movement proteins (Wu and Cheng, 2020).

Movement proteins of the *Tobamovirus* genus interact with SYNAPTOTAGMIN A (SYTA), a resident of the plasma membrane–endoplasmic reticulum contact sites, and trigger relocalization of SYTA to the PD (Yuan et al., 2018). The MP–SYTA complex causes remodeling of the PD to establish virus replication sites and changes the PD permeability, thus enabling virus cell-to-cell movement (Levy et al., 2015). In the *syta* mutant, intercellular movement is reduced not only for tobamoviruses, but also for viruses from the *Begomovirus* and *Potyvirus* genera, suggesting a general proviral role of SYTA (Lewis and Lazarowitz, 2010; Uchiyama et al., 2014; Cabanillas et al., 2018).

At the endoplasmic reticulum, TGBp2 protein of *Potato virus X* (*Potexvirus*) induces the formation of mobile vesicles that sequentially recruit viral replication complexes, TGBp3 and TGBp1, resulting in the formation of mobile VROs (mVROs) (Ju et al., 2005; Samuels et al., 2007; Tilsner et al., 2013; Wu et al., 2019a). Intracellular transport of mVROs towards the plasmodesmata is likely mediated by microfilaments, indicated by the TGBp2 colocalization with actin and the need of intact actin filaments for efficient intercellular movement (Ju et al., 2005; Harries et al., 2009). At the plasmodesmata, the three TGB movement proteins orchestrate formation of replication compartments that functionally relate to perinuclear X-bodies described in the previous subsection. The TGBp2 and TGBp3 are responsible for remodeling ER membranes at the PD orifice (Tilsner et al., 2013; Wu et al., 2019a). The necessary modification of the PD size exclusion limit is likely mediated by TGBp2, which is capable of inducing ER tubule constrictions in a reticulon-like manner (Lazareva et al., 2021). Specific antibody-based labelling of the PVX virions in the plasmodesmata connecting infected cells elegantly demonstrated that virions represent the probable infectious entity in the cell-to-cell transport (Santa Cruz et al., 1998). The multifunctional TGBp1 protein is essential for loading the PVX virions into the plasmodesmal cavity (Tilsner et al., 2013).

The *Turnip mosaic virus* (*Potyvirus*) cell-to-cell movement depends on the 6K2 motile VROs, indicated by its inhibition by brefeldin A and concanamycin A, compounds that disturb pre- and post-Golgi trafficking pathways (Agbeci et al., 2013). The TuMV intercellular movement is also inhibited by actin-depolymerizing drugs and the expression of a dominant-negative mutant of myosin XI-2, demonstrating that the 6K2 VRO trafficking is mediated by actin filaments (Agbeci et al., 2013). The TuMV movement protein, P3N-PIPO, guides the cylindrical inclusion (CI) viral protein to plasmodesmata, where CI forms conical structures that are essential for intercellular movement (Wei et al., 2010b). Due to a polymerase slippage within the P3 cistron, P3N-PIPO is a chimeric protein that consists of N-terminal portion of P3 protein and a small ORF termed PIPO, for Pretty Interesting *Potyviridae* ORF (Rodamilans et al., 2015). The multipartite interactions among 6K2, P3, P3N-PIPO and CI are indispensable for recruitment of the 6K2 motile VROs to the PD (Chai et al., 2020). However, the identity of the infectious material that is transferred between the adjacent cells remains an unresolved issue. The coat protein (CP) deletion or point mutations within its core domain abolished the TuMV intercellular movement but they did not affect viral replication (Dai et al., 2020). The observed requirement of the native coat protein for efficient cell-to-cell movement suggests that the transported infectious entities may be virions (Dai et al., 2020; Wang, 2021). In contrast, detection of 6K2 intercellular movement implies that the viral RNA complex may be transported in a membrane-mediated manner (Grangeon et al., 2013). Using an infection-free movement assay, CI and P3N-PIPO were identified as sufficient to support the intercellular movement of the 6K2-induced vesicles (Movahed et al., 2017). The 6K2 cell-to-cell movement was abolished when CI carried a mutation in the 6K2-interacting domain, indicating that CI serves as a docking platform for the 6K2 vesicles (Movahed et al., 2017). In a similar way, the 6K2 replication vesicles, released into the extracellular space during membrane fusion of multivesicular bodies with the plasma membrane, may represent vehicles for systemic TuMV infection (Wan et al., 2015b; Movahed et al., 2019a). To unite these contradictory models of the TuMV cell-to-cell movement, we may hypothesize that the PD-localized coat protein may represent an essential factor in the movement of the 6K2-containing replication vesicles through plasmodesmata, presumably by interaction with certain components of the transported vesicles. Further analyses of the coat protein function at plasmodesmata may resolve this puzzling issue. Interestingly, the TuMV multifunctional cylindrical inclusion (CI) protein and the viral genome-linked protein (VPg), which are essential for intercellular movement, also participate in viral replication and are retrieved from the plasma membrane to endosomes by clathrin-mediated endocytosis. This uptake of viral proteins is regulated by the DYNAMIN-RELATED PROTEIN family of GTPases that assists in the endocytic vesicle budding (Wu et al., 2018). The expression of a dominant-negative form of DRP1A suppresses TuMV replication, indicating that the dynamic partitioning of CI and VPg between plasma membrane and early/late endosomes likely represents an important regulatory mechanism (Wu et al., 2020). The CI and VPg viral proteins are recognized by the β subunit of the AP2 clathrin adaptor complex, as the corresponding *ap2β* knockout impairs intracellular endosomal trafficking of both viral cargoes and inhibits TuMV replication (Wu et al., 2020). In more detail, the endocytic uptake of CI depends on the interaction of its dileucine motif with the AP2β subunit (Wu et al., 2022b). Similar endocytosis-assisted cycling of viral proteins between the plasma membrane and early endosomes has been reported also for movement proteins of *Ourmia melon virus* (OuMV, +ssRNA, genus *Ourmiavirus*) and *Cauliflower mosaic virus* (CaMV, dsDNA, genus *Caulimovirus*) (Carluccio et al., 2014; Ozber et al., 2022).

**Figure 1:**
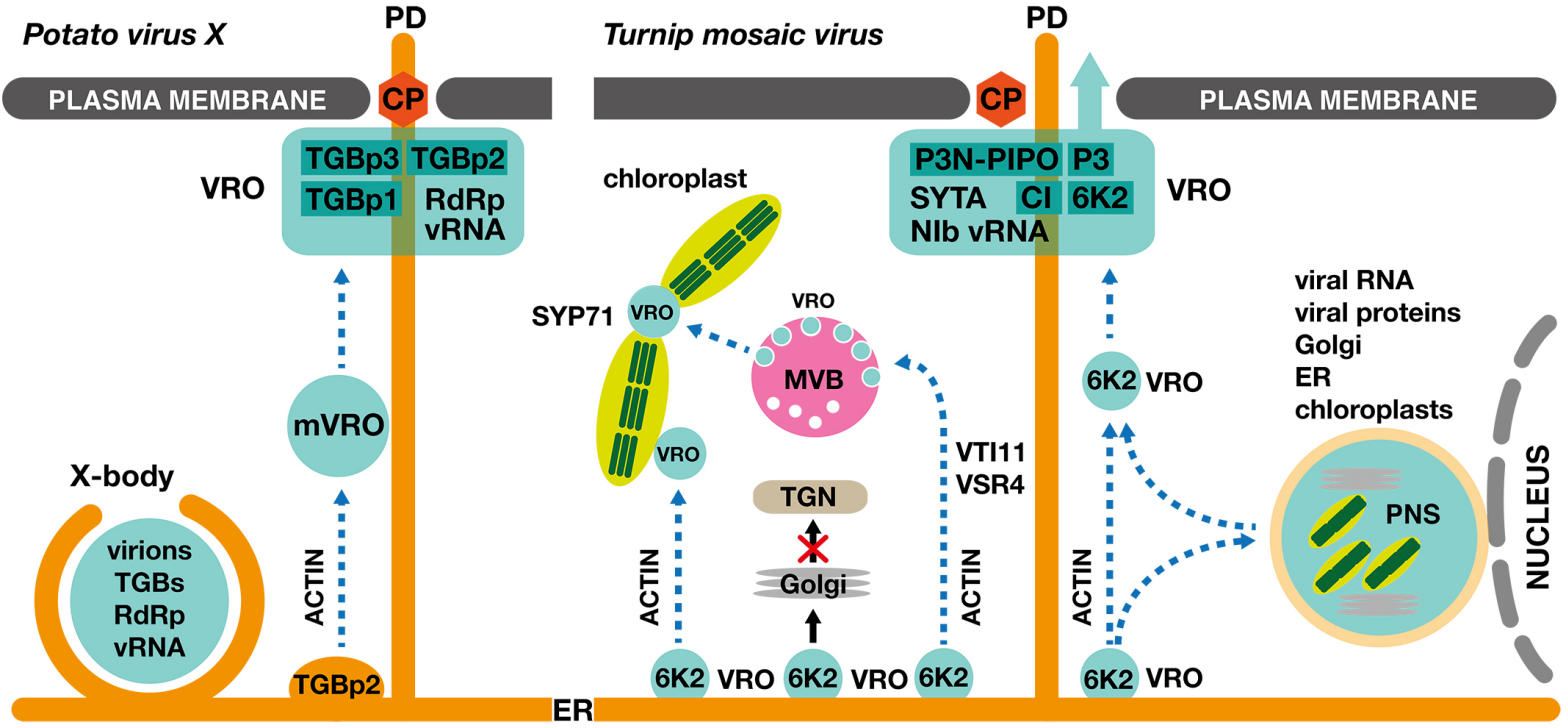
Establishment of viral replication organelles and viral cell-to-cell movement in PVX- and TuMV-infected plant cells. Left panel: *Potato virus X* (*Potexvirus*) induces formation of an intricate perinuclear replication compartment called the X-body, containing triple gene block proteins (TGBp1, TGBp2 and TGBp3), viral RNA (vRNA) and viral RNA-dependent RNA polymerase (RdRp). In addition, ER-derived TGBp2 vesicles establish mobile viral replication organelles (mVROs) that contribute to the biogenesis of a static replication compartment localized to the plasmodesmata for efficient viral intercellular movement, mediated by the PVX coat protein (CP). The intracellular movement of the TGBp2 vesicles and the mVROs is mediated by the actomyosin system. Right panel: ER-derived viral replication organelles of *Turnip mosaic virus* (*Potyvirus*), labeled with 6K2 viral protein, form secondary VROs at chloroplasts and multivesicular bodies (MVBs). The formation of the interplastidial VROs requires SYP71, a plant-specific Qc-SNARE. The 6K2-containing VROs, which enter the Golgi apparatus *via* a conventional COPII-mediated anterograde transport pathway, represent a nonproductive population. The TuMV-infected cell contains a large static perinuclear structure (PNS), which comprises amalgamated Golgi stacks, the endoplasmic reticulum, chloroplasts, and viral replication machinery. The PNS receives but also releases motile VROs. The 6K2-labelled motile VROs also contribute to the formation of dynamic replication compartments in the proximity of the plasmodesmata, containing viral RNA (vRNA), viral RNA-dependent RNA polymerase (NIb), viral movement protein (P3N-PIPO), viral protein P3, cylindrical inclusion viral protein (CI) and the host SYNAPTOTAGMIN A (SYTA). Cell-to-cell movement can be mediated either by virions containing the coat protein (CP) or by direct movement of the 6K2-containing VRO through plasmodesmata. The intracellular movement of the 6K2 VROs is mediated by the actomyosin system.

In conclusion, positive-strand RNA viruses are master manipulators of the endomembrane system in plants. They appropriate not only existing host endomembrane compartments but also other organelles, such as chloroplasts or peroxisomes, to establish intricate viral replication organelles (**Figure 1**). To do so, they hijack numerous host proteins involved in membrane shaping or fusion, and activate host cellular pathways, including the unfolded protein response, to successfully perform their replication.

## 3 Plant viruses and membrane-mediated immunity

In this section, we discuss a paradigm shift regarding the role of membrane-localized receptor-like kinases, their associated proteins and the subsequent signaling cascades in plant antiviral immunity. In continuation, we discuss two important questions - what molecules may represent the viral molecular patterns that are recognized by these receptor complexes and where may this recognition take place.

### 3.1 Membrane-localized pattern recognition receptor complexes

Viruses are obligate intracellular parasites and plant antiviral immunity was accordingly thought to be mediated mainly by RNA interference and intracellular effector-triggered immunity (Nicaise, 2014). Therefore, detection of extracellular pathogen- and danger-associated molecular patterns (PAMPs and DAMPs) was not considered noteworthy for virus pathogenesis. This detection is mediated by cell surface-localized pattern recognition receptors (PRRs) that are represented by either receptor-like kinases (RLKs) or receptor-like proteins (RLPs) (He et al., 2018). Both types of receptors recognize PAMPs or DAMPs *via* diverse extracellular domains and require assistance of co-receptors for signal transduction, resulting in calcium (Ca^2+^) influx, reactive oxygen species burst, activation of calcium-dependent kinases (CDPKs) and mitogen-activated protein kinases (MAPKs), hormone signaling, and changes in gene expression, all directed at confinement of the infection (Tang et al., 2017; Zhang and Zhang, 2022). This extracellular signal-based response is known as the pattern-triggered immunity (PTI). In the last decade, however, it was demonstrated that mutants in SOMATIC EMBRYOGENESIS RECEPTOR-LIKE KINASEs (SERKs) show increased susceptibility to several RNA viruses. SERKs represent quintessential components of pattern-triggered immunity and function as coreceptors of the PRR proteins (Ma et al., 2016). Not surprisingly, the discovery of their involvement in plant antiviral responses initiated search for a novel model of plant antiviral immunity. Arabidopsis plants lacking SERK3, better known as BRI1-ASSOCIATED KINASE 1 (BAK1), or SERK4, also known as BAK1-LIKE 1 (BKK1), showed enhanced susceptibility to *Turnip crinkle virus* (TCV, +ssRNA, genus *Betacarmovirus*) infection (Yang et al., 2010). Higher viral accumulation and classical virus-induced symptoms were also detected in the *bak1* Arabidopsis mutant plants infected with *Tobacco mosaic virus* (*Tobamovirus*) and *Oilseed rape mosaic virus* (ORMV, +ssRNA, genus *Tobamovirus*) (Kørner et al., 2013). For some viral species, BAK1 and BKK1 may contribute redundantly to antiviral immunity, as it was demonstrated for *Plum pox virus* (PPV, +ssRNA, genus *Potyvirus*). Only Arabidopsis plants carrying both *bak1* and *bkk1* mutations displayed a massive increase in PPV accumulation (Nicaise and Candresse, 2017).

During bacterial and fungal infections, the key PTI proteins are targets of intracellular effectors that aim to shut down the associated defense pathways. It is conceivable that plant viruses may employ a similar *modus operandi*. Indeed, the first evidence came from *in planta* expression of the PPV capsid protein (CP), which was found to strongly impair PTI responses. The induction of the bacterial flagellin-induced marker genes was suppressed in CP-expressing samples in both *N. benthamiana* and Arabidopsis (Nicaise and Candresse, 2017). PTI-suppressing activity was also detected for the movement protein of *Cucumber mosaic virus* (*Cucumovirus*). Expression of the CMV movement protein in Arabidopsis and *N. benthamiana* suppresses reactive oxygen species (ROS) production triggered by multiple pathogen-associated molecular patterns (PAMPs), including bacterial flagellin and EF-Tu, and fungal-derived chitin (Kong et al., 2018).

Infection of *Tomato yellow leaf curl China virus* (*Begomovirus*) induces the activation of a host mitogen-activated protein kinase (MAPK) cascade, which represents a characteristic PTI readout. To counter these responses, the βC1 protein of TYLCCNV interacts with MKK2 and MPK4 and inhibits their kinase activity (Hu et al., 2019). A coat protein of *Beet black scorch virus* (*Betanecrovirus*) was also identified as a potent inhibitor of MAPK-mediated antiviral defense. The viral effector does not bind the kinase itself but interferes with the binding of 14-3-3a protein to MAPKKKα, resulting in the MAPKKKα protein instability (Gao et al., 2022). The molecular role of MAPK cascade in suppression of viral infections was demonstrated for *Barley yellow striate mosaic virus* (BYSMV, -ssRNA, genus *Cytorhabdovirus*). Barley MPK3 interacts with the BYSMV nucleoprotein (N) and phosphorylates it, causing formation of unstable N–RNA complexes and subsequently abolishing virus infection (Ding et al., 2022).

In addition to virus-triggered classic PTI responses listed above, the plant antiviral defense seems to involve an additional strategy. This novel layer of the antiviral defense depends on the transmembrane receptor-like kinase NSP-INTERACTING KINASE 1 (NIK1). Tomato and soybean NIK1 proteins were first identified as interacting partners of the nuclear shuttle proteins (NSP) of bipartite geminiviruses *Tomato golden mosaic virus* (TGMV, ssDNA, genus *Begomovirus*) and *Tomato crinkle leaf yellows virus* (TCrLYV, ssDNA, genus *Begomovirus*) through yeast two-hybrid assays and *in vitro* protein binding experiments (Mariano et al., 2004). Arabidopsis genome encodes three homologs, designated NIK1, NIK2 and NIK3 (Fontes et al., 2004). Taking advantage of genetic tools available for Arabidopsis and the fact that bipartite geminivirus *Cabbage leaf curl virus* (CaLCuV, ssDNA, genus *Begomovirus*) infects Arabidopsis, it was shown that binding of the CaLCuV NSP to NIKs inhibited their kinase activity and that *nik* single mutant plants were susceptible to geminivirus infection (Fontes et al., 2004). It was demonstrated that the kinase domain of NIK1 binds Arabidopsis RIBOSOMAL PROTEIN L10 A (rpL10A) (Rocha et al., 2008). The NIK1-dependent phosphorylation promotes translocation of rpL10A to the nucleus (Carvalho et al., 2008), where it interacts with a MYB-like protein, L10-INTERACTING MYB DOMAIN-CONTAINING PROTEIN (LIMYB). These two proteins in concert fully repress the expression of *RIBOSOMAL PROTEIN* (*RP*) genes, resulting in decreased viral RNA association with polysome fractions and enhanced tolerance to geminivirus infection (Zorzatto et al., 2015). The C4 protein of monopartite geminivirus *Tomato yellow leaf curl virus* (TYLCV, ssDNA, genus *Begomovirus*) can broadly interact with various subfamilies of plant receptor-like kinases (RLKs) (Garnelo Gómez et al., 2019), suggesting that C4 of a monopartite begomovirus may exert the NSP role. Given the structural similarity of NIK proteins to SERKs (Teixeira et al., 2019), it is tempting to speculate that NIKs may function as coreceptors of yet-unidentified receptors of virus-associated molecular patterns. Supporting this hypothesis, NIK1 was found to interact with the bacterial flagellin receptor FLS2. The NIK1-overexpressing Arabidopsis plants show enhanced susceptibility to bacterial infections, likely caused by the NIK1 interference with the formation of a functional FLS2 complex (Li et al., 2019).

### 3.2 Virus-associated molecular patterns

The BAK1-dependent responses were observed upon induction with crude extracts from tobamovirus infected *N. benthamiana* plants but not with purified virions, indicating that some internal viral components or replication/transcription intermediates, such as proteins or certain types of nucleic acids, may be responsible for the PTI induction (Kørner et al., 2013). In addition, viral nucleic acid extracts from *Cabbage leaf curl virus*-infected Arabidopsis plants were able to activate NIK1-mediated responses. Using *nik1* and *nik2* single and double mutant plants, it was also demonstrated that this elicitation is dependent on NIK1 and/or NIK2 (Teixeira et al., 2019).

Double-strand RNAs (dsRNAs) can be generated in life cycles of ssRNA, dsRNA and DNA viruses and may thus represent general molecular patterns recognized by the antiviral PTI machinery. In mammalian cells, dsRNA is recognized by the endosomal TLR3 Toll-like receptor that triggers antiviral cellular responses (Lester and Li, 2014). It was proposed that fusion of the autophagosome containing apoptotic cell debris with the TLR3-containing endosome allows viral dsRNA recognition (Fitzgerald and Kagan, 2020). In plants, purified dsRNAs from the *Oil seed rape mosaic virus*-infected plants and the synthetic dsRNA analog polyinosinic–polycytidylic acid (poly(I:C)) triggered typical PTI responses, such as activation of MAP kinases and accumulation of pathogenesis-related phytohormones (Niehl et al., 2016). Ethylene production, a well-known marker of PTI, was impaired only in the *serk1* mutants, but not in *bak1/serk3* a *bkk1/serk4* plants, suggesting a putative specificity within the SERK family in antiviral responses. Importantly, the PTI responses listed above were observed in plants lacking the two major DICER-LIKE proteins, indicating that dsRNA represents a *bona fide* viral PAMP that functions independently from the RNAi pathway (Niehl et al., 2016). Strikingly, the poly(I:C) treatment of *N. benthamiana* infected with *Tobacco mosaic virus* reduced viral cell-to-cell movement, while viral replication and accumulation remain unaffected (Huang et al., 2023). Subsequent analyses revealed that the poly(I:C) molecular pattern is recognized by a receptor complex that contains SERK1 and the signal is further transmitted by receptor-like cytoplasmic kinases BIK1 and PBL1, leading to callose deposition at plasmodesmata and thus decreased viral intercellular movement. Interestingly, the poly(I:C) induced callose deposition is inhibited by movement proteins derived from +ssRNA tobamoviruses *Tobacco mosaic virus*, *Oilseed rape mosaic virus* and *Turnip vein clearing virus*, but the exact molecular mechanism of such inhibition is currently unknown (Huang et al., 2023). It remains to be tested whether other members of the SERK protein family also participate in the dsRNA sensing or whether their corresponding receptor complexes detect other yet-unidentified viral molecular patterns.

Considering that mammalian TLR signal presence of foreign nucleic acids from endosomal compartments (Tan et al., 2018), can plant virus-specific PRRs also function from endosomes? Plant receptors of bacterial PAMPs, such as FLS2 or EFR (receptor of bacterial elongation factor EF-Tu), internalize into endosomal compartments only after ligand perception (Mbengue et al., 2016), unlike structurally-similar brassinosteroid receptor BRI1 that cycles between the PM and endosomes independently of its ligand (Geldner et al., 2007). Interestingly, observed endocytosis of FLS2 seems crucial for certain PTI processes, such as callose deposition and stomatal closure, but not for others, including activation of MAP kinases or production of reactive oxygen species, suggesting that FLS2 may activate certain responses after its internalization into endosomal membranes (Mbengue et al., 2016). It was recently reported that external application of nonself nucleic acids resulted in their rapid internalization by endocytosis in Arabidopsis (Chiusano et al., 2021). Such observation provides a suitable explanation for the previously reported PTI activation by externally applied dsRNAs, poly(I:C) and viral extracts (Kørner et al., 2013; Niehl et al., 2016). In addition, NSP from *Cabbage leaf curl virus* (*Begomovirus*) was found to interact not only with NIKs but also with a novel SNARE-like protein, designated NSP-INTERACTING SYNTAXIN DOMAIN-CONTAINING PROTEIN (NISP). NISP, which harbors a syntaxin-6 domain at the N-terminus and a transmembrane segment at the C-terminus, exhibits a proviral function as the *nisp1* mutant is less susceptible to begomovirus infection. Using fluorescent molecular markers and FM4-64 labeling, NISP-GFP was localized to early endosomes (Gouveia-Mageste et al., 2021). These findings suggest that NISP may function in recruitment of begomoviral NSPs to the early endosomal membrane where they may, among other functions, inhibit the NIK-containing PRR complexes.

**Figure 2:**
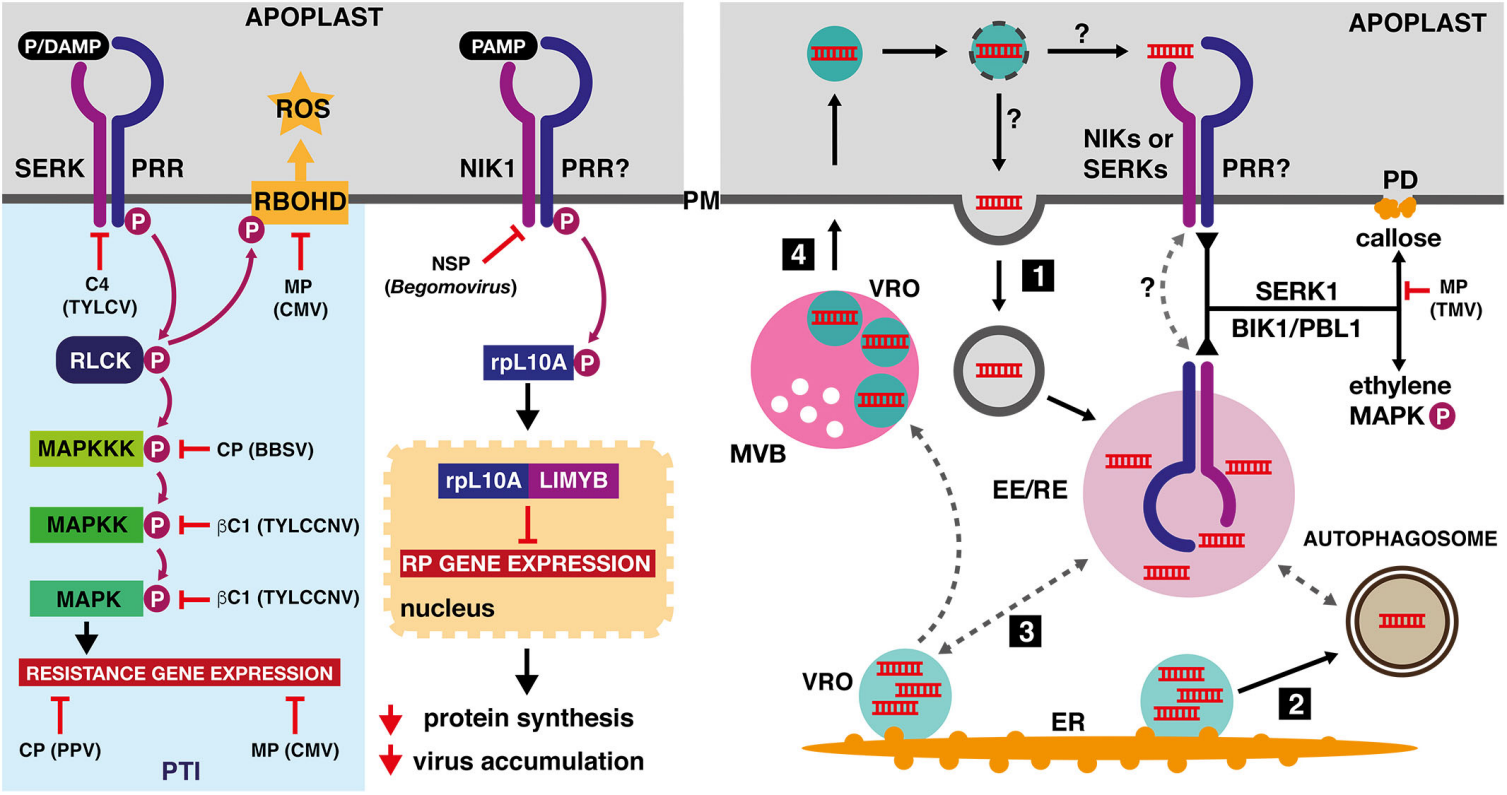
Effects of viral proteins on pathogen-triggered immunity and possible ways of viral dsRNA sensing in plants. Left panel: The coat protein (CP) of *Plum pox virus* (PPV, *Potyvirus*) and the movement protein (MP) of *Cucumber mosaic virus* (CMV, *Cucumovirus*) repress transcriptional reprogramming induced by pattern-triggered immunity (PTI). The C4 protein of *Tomato yellow leaf curl virus* (TYLCV, *Begomovirus*) and the nuclear shuttle protein (NSP) of various *Begomovirus* species inhibit membrane-localized receptor-like kinases from the SERK and NIK families. Activation of the NIK1-containing receptor complex leads to binding of phosphorylated ribosomal protein rpL10A to MYB-like transcription factor (LIMYB). The resulting protein complex represses expression of ribosomal protein genes, leading to an enhanced tolerance to viral infection. The coat protein of *Beet black scorch virus* (BBSV, *Betanecrovirus*) and the βC1 virulence factor of *Tomato yellow leaf curl China virus* (TYLCCNV, *Begomovirus*) are inhibitors of MAPK-mediated defense processes. The CMV movement protein was identified as a potent suppressor of the reactive oxygen species (ROS) production, mediated by plasma-membrane localized RESPIRATORY BURST OXIDASE HOMOLOGUE D (RBOHD). Right panel: PRR complexes, which are localized to the early or recycling endosomes (EE/RE) or cycle between the EE/RE and the plasma membrane, may detect dsRNA that was endocytosed from the extracellular space (1), delivered to the certain endosome population by means of autophagosomes (2) or by contact with the viral replication organelles (3). Alternatively, plasma membrane-localized PRR complexes may detect dsRNA delivered to the apoplast by extracellular vesicles, derived from intralumenal secondary replication organelles formed by motile ER-derived VROs in the prevacuolar compartments of the TuMV-infected plant cells (4). The viral PAMP signal, represented by dsRNA and detected by the SERK1-containing receptor complex, is further transmitted by receptor-like cytoplasmic kinases (RLCKs) BIK1 and PBL1, resulting in callose deposition, ethylene production and MAPK activation. The callose deposition is inhibited by the movement proteins (MP) of *Tobacco mosaic virus* (TMV) and other tobamoviruses.

In conclusion, we hypothesize that viral PAMPs, likely represented by double-strand RNA molecules and certain viral proteins, are recognized by the yet-unidentified membrane-localized receptor complexes. After detection, the PRR proteins and their coreceptors from the SERK family initiate classic PTI responses, including ethylene production, callose deposition and MAP kinase activation. Alternatively, the receptor complexes containing the NIK1 family of putative coreceptors trigger general translational attenuation, leading to decreased virus accumulation. The viral PAMP-specific receptor complexes may be localized to endosomal compartments, in a topology analogous to the dsRNA-specific receptors in metazoans, but the virus-associated molecular patterns may be recognized also in the extracellular space, in a similar manner as the bacterial and fungal PAMPs (**Figure 2**). The recently identified MVB-derived vesicles, released during *Turnip mosaic virus* (*Potyvirus*) infection into the apoplast in Arabidopsis and *N. benthamiana*, contain viral nucleic acids and proteins, and may thus facilitate detection of viral PAMPs by their corresponding plasma membrane-localized receptors (Movahed et al., 2019a).

## 4 Autophagy in plant virus interactions

In this section, we will present selective autophagy as a critical component of plant antiviral defense processes. We will also discuss how viruses utilize autophagy as a proviral tool to selectively target and degrade functional host factors to evade plant defense responses.

### 4.1 Antiviral functions of selective plant autophagy

Autophagy facilitates the vacuole-dependent unspecific (bulk) or highly selective degradation of cellular components, mediated by conserved AUTOPHAGY-RELATED (ATG) proteins (Marshall and Vierstra, 2018). A membrane structure named phagophore is formed upon activation of autophagy and progresses into a double-membrane compartment, containing the autophagy cargo, known as the autophagosome (Wun et al., 2020). In plants, autophagy activation is positively regulated by the SUCROSE NON-FERMENTING1-RELATED PROTEIN KINASE1 (SnRK1) protein family and repressed by the TARGET OF RAPAMYCIN complexes (Soto-Burgos et al., 2018). The Arabidopsis ATG1–ATG13 kinase complex is required for the phagophore initiation and represents both the regulator and the target of autophagic recycling, thus allowing the plant cell to reset the phagophore initiation process (Suttangkakul et al., 2011). The ATG1–ATG13 complex promotes ATG9–ATG2–ATG18-mediated delivery of lipids to the growing phagophore (Zhuang et al., 2017). Simultaneously, the phagophore membrane is enriched in phosphatidylinositol 3-phosphate by the phosphatidylinositol 3-kinase protein complex (Liu et al., 2020). The ubiquitin-fold protein ATG8 is conjugated to phosphatidylethanolamine by the ATG12–ATG5–ATG16 complex and this lipidation allows for its phagophore membrane insertion (Chung et al., 2010). At the engulfing phagophore, the ATG8–PE conjugate acts as a tethering platform for the selective autophagy. ATG8 can bind autophagy cargo directly or *via* a range of the ATG8-interacting motif (AIM)-containing proteins that function as highly selective autophagy receptors (Liu et al., 2021). While bulk autophagy does not require actin filaments, the selective autophagy receptors colocalize with actin, suggesting that the selective autophagy cargo needs to be actively transported and concentrated at the phagophore (Zientara-Rytter and Sirko, 2014; Zheng et al., 2019). Subsequently, the double membrane structure is sealed by the ESCRT proteins (Zhou et al., 2019). The autophagosome eventually fuses with the tonoplast, releasing its content for degradation by vacuolar hydrolases (Gao et al., 2015; Kalinowska and Isono, 2018). However, the direct fusion of the autophagosome and the vacuole has been recently challenged. It was shown that the interaction of the autophagosome-localized CELL DEATH RELATED ENDOSOMAL FYVE/SYLF PROTEIN 1 (CFS1) adaptor with an MVB-localized subunit of the ESCRT complex has a crucial role in the autophagic flux, as the delivery of autophagosomes to the vacuole is disrupted in the *cfs1* Arabidopsis plants (Sutipatanasomboon et al., 2017; Zhao et al., 2022). It was proposed that CFS1 mediates formation of plant amphisomes, which were previously identified in metazoans, and that plant amphisomes may serve as sorting hubs for multivesicular bodies and autophagosomes before fusing with the vacuole (Zhao et al., 2022).

The first direct interaction of a viral protein and the selective plant autophagy machinery was demonstrated for the βC1 virulence factor of *Cotton leaf curl Multan virus* (CLCuMuV, ssDNA, genus *Begomovirus*) (Haxim et al., 2017). Using a yeast two-hybrid approach (Y2H), pull-down assays and bimolecular fluorescence complementation (BiFC) experiments, ATG8f proteins from tomato (*Solanum lycopersicum*) and tobacco (*Nicotiana benthamiana*) were identified as βC1 interacting partners. The specific interaction between ATG8f and βC1 was attributed to an 11-amino acid motif within the βC1 viral protein. A mutation in this motif resulted in higher accumulation of CLCuMuV in tobacco. Silencing autophagy-related genes *ATG5* and *ATG7* caused reduced plant resistance to begomoviruses *Cotton leaf curl Multan virus*, *Tomato yellow leaf curl virus* and *Tomato yellow leaf curl China virus*, whereas activating autophagy led to enhanced plant resistance (Haxim et al., 2017). The C1 nucleoprotein of *Tomato leaf curl Yunnan virus* (TLCYnV, ssDNA, genus *Begomovirus*) is also degraded by autophagy *via* a direct interaction with ATG8 (Li et al., 2020b). This interaction, mediated by the AIM motif in the C1 protein, was demonstrated using the *ATG8h* coding sequence from *S. lycopersicum* in both the Y2H assay and the BiFC experiments. Silencing *ATG8h*, *ATG5*, and *ATG7* genes in *S. lycopersicum* and *N. benthamiana* inhibits the degradation of C1, which in turn promotes TLCYnV infection. Importantly, karyopherin EXPORTIN1 (XPO1) from tomato and tobacco is necessary for the transfer of the C1-ATG8h complex from the nucleus to the cytoplasm to facilitate the C1 degradation (Li et al., 2020b). Another direct interaction with ATG8 was reported for a movement protein of *Citrus leaf blotch virus* (CLBV, +ssRNA, genus *Citrivirus*) (Niu et al., 2021). The identified AIM motif within the CLBV MP interacts specifically with the ATG8C1 isoform, while additional binding domains at the N-terminus of the CLBV MP were hypothesized for an interaction with the ATG8i isoform in tobacco (Niu et al., 2021). The requirements of a particular ATG8 isoform for a successful degradation of distinct viral proteins suggests a coevolution-driven selectivity.

However, autophagy in plants involves not only direct targeting of the viral component by the core autophagy protein ATG8 but mainly requires cargo receptors as intermediaries. A central role plays an autophagy receptor NEXT TO BRCA1 GENE 1 (NBR1)/JOKA2, which selectively brings the autophagy cargo to the phagophore through interaction with ATG8. NBR1-dependent selective autophagy promotes degradation of the non-assembled virus capsid protein P4 of *Cauliflower mosaic virus* (*Caulimovirus*), which restricts viral infection in Arabidopsis (Hafrén et al., 2017). Upon CaMV infection, Arabidopsis *atg5* and *atg7* autophagy-defective mutants exhibit more severe symptoms than wild-type plants and accumulate higher amounts of the P4 capsid protein, while the levels of other viral proteins remain unaltered. In a typical tug-of-war strategy, the CaMV P6 protein sequesters viral particles to inclusion bodies, where they are protected from the autophagy machinery (Hafrén et al., 2017). NBR1-mediated selective autophagy has been also shown to target the RNA silencing suppressor (RSS) helper-component protease HCpro from TuMV (Hafrén et al., 2018). Arabidopsis autophagy mutants show stronger TuMV disease symptoms including severe stunting and accelerated leaf senescence, compared to the wild type plants. Intriguingly, the TuMV seems to counteract NBR1-dependent autophagy during infection using other viral proteins, thereby limiting its antiviral potential (Hafrén et al., 2018). The silencing suppressors such as HCpro of *Tobacco etch virus* (TEV, +ssRNA, genus *Potyvirus*) or 2b from *Cucumber mosaic virus* (*Cucumovirus*) or *Tomato aspermy virus* (TAV, +ssRNA, genus *Cucumovirus*) are not recognized by NBR1, but instead bind calmodulin-like protein rgs-CaM and the resulting protein complex is subsequently degraded by autophagy (Nakahara et al., 2012). The unambiguous role of autophagy in the HCpro–rgs-CaM complex degradation is indicated by its sensitivity to 3-methyladenine, which represents a potent inhibitor of the autophagosome biogenesis (Nakahara et al., 2012). A recently identified autophagy cargo receptor, P3 INTERACTING PROTEIN in *N. benthamiana* (NbP3IP), facilitates the degradation of the P3 protein, an RNA silencing suppressor encoded by the *Rice stripe virus* (*Tenuivirus*) (Jiang et al., 2021). Using BiFC and pull-down assays, it was demonstrated that NbP3IP interacts with NbATG8f. While silencing *ATG5* and *ATG7* in *N. benthamiana* promoted RSV infection, tobacco plants with activated autophagy contained lower amounts of virus (Jiang et al., 2021).

ATG6/BECLIN1 represents another potential cargo receptor, interacting with NIb, the TuMV RNA-dependent RNA polymerase (RdRp) (Li et al., 2018). Deficiency of ATG6 or ATG8a in *N. benthamiana* increases NIb accumulation and promotes viral infection. A noteworthy aspect is that ATG6 also interacts with RdRps from other potyviruses, such as *Plum pox virus*, *Soybean mosaic virus*, and *Tobacco etch virus*, as well as RdRps from *Cucumber green mottle mosaic virus* (CGMMV, +ssRNA, genus *Tobamovirus*) and *Pepino mosaic virus* (PepMV, +ssRNA, genus *Potexvirus*) (Li et al., 2018). These findings suggest that the ATG6-mediated targeting of RdRps could be a general mechanism for restricting viral infection in plants. However, a recent report challenged the strict antiviral role of selective autophagy in TuMV infection and suggested an additional function in promoting viral replication (Li et al., 2020a).

### 4.2 Plant autophagy in promoting viral infection

The proviral role of receptor-independent bulk autophagy was proposed to be linked to the suppression of virus-associated senescence that leads to a significant extension of the virus production timespan and to better chances for virus particle acquisition by aphid vectors (Hafrén et al., 2017). However, an increasing number of reports identified a proviral role of selective autophagy. A small viral membrane protein 6K2 of *Turnip mosaic virus*, described in the previous section as a key protein in the formation of viral replication compartments, upregulates the *NBR1* gene expression through the IRE1/bZIP60 pathway. It was demonstrated that overexpression of NBR1 stimulates TuMV replication, while its deficiency inhibits virus infection (Li et al., 2020a). The upregulated NBR1 competes with BECLIN1 for interaction with NIb, the TuMV RNA polymerase, and binds ATG8f of both *Nicotiana benthamiana* and Arabidopsis. The resulting NIb–NBR1–ATG8f complex colocalizes with 6K2 to the vacuolar membrane. At the tobacco tonoplast, ATG8f interacts with TONOPLAST INTRINSIC PROTEIN 1 (NbTIP1), resulting in the formation of numerous viral replication complexes (Li et al., 2020a). An interesting example of proviral autophagy functions was shown to be mediated by P0 protein from *Turnip yellows virus* (TuYV, +ssRNA, genus *Polerovirus*). P0 is an F-box protein that interacts with S PHASE KINASE-ASSOCIATED PROTEIN 1 (SKP1), a core component of the SKP1–CULLIN1–F-box protein (SCF) E3 ubiquitin ligase complex (Bortolamiol et al., 2007). The SCF complex ubiquitinates ARGONAUTE1 (AGO1), a key component of the RNA-induced silencing complex (RISC) in Arabidopsis, but the AGO1 ubiquitination does not cause its 26S proteasome degradation (Baumberger et al., 2007). Instead, ubiquitinated AGO1 and P0 interact with ATG8-INTERACTING PROTEIN1 (ATI1) at the endoplasmic reticulum and the resulting protein complex is degraded by ATG5/ATG7-mediated autophagy (Michaeli et al., 2019).

**Figure 3:**
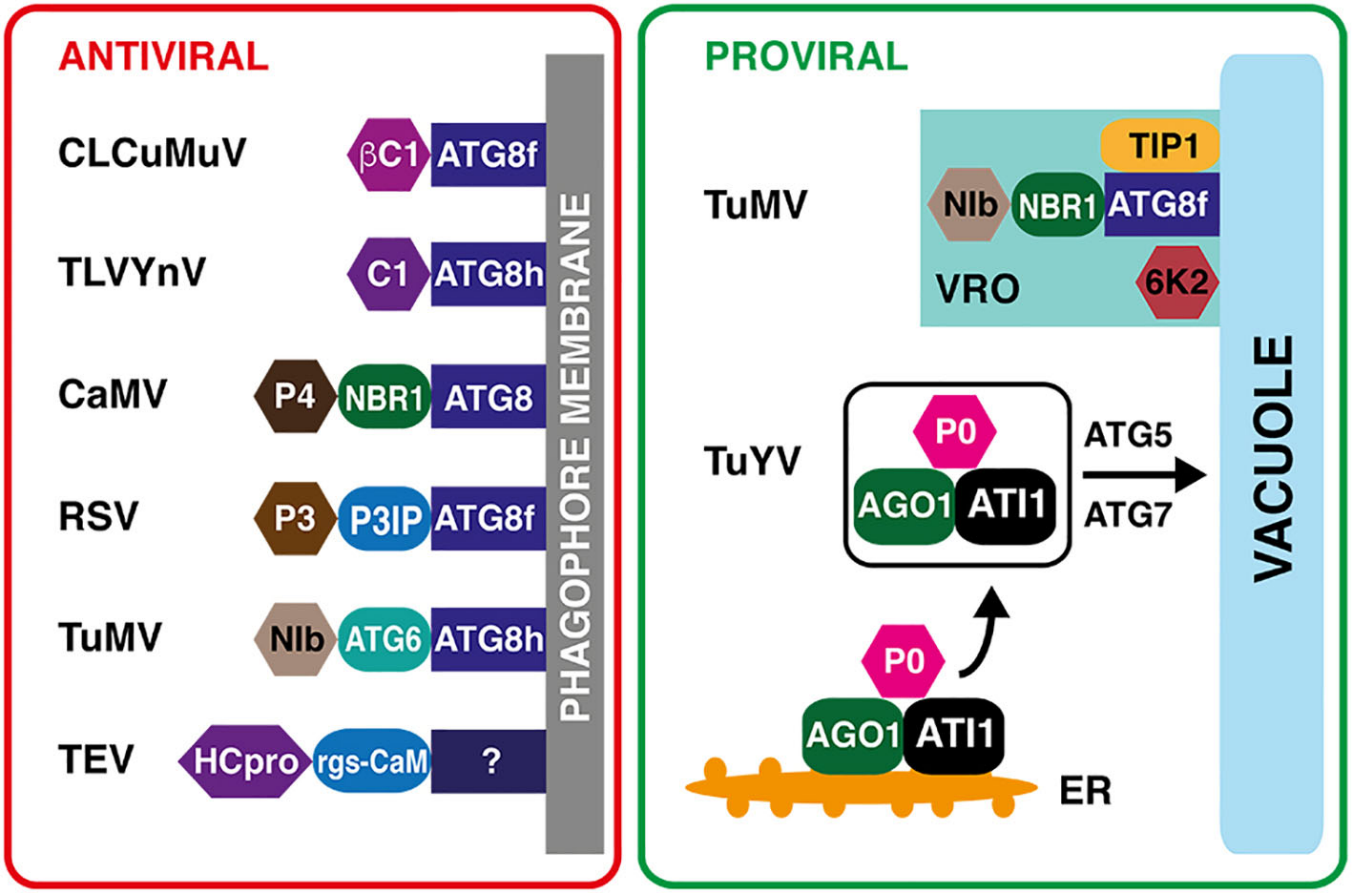
Proviral and antiviral roles of plant selective autophagy. Left panel: Virus–host protein interactions with the antiviral outcome. The βC1 virulence factor of *Cotton leaf curl Multan virus* (CLCuMuV, *Begomovirus*) and the C1 nucleoprotein of *Tomato leaf curl Yunnan virus* (TLCYnV, *Begomovirus*) are recognized directly by the phagophore-localized ATG8 protein. The non-assembled virus capsid protein P4 of *Cauliflower mosaic virus* (CaMV, *Caulimovirus*) is recognized by the NEXT TO BRCA1 GENE 1 (NBR1) specific autophagy receptor. The P3 INTERACTING PROTEIN (P3IP) autophagy cargo receptor binds the P3 RNA silencing suppressor of *Rice stripe virus* (RSV, *Tenuivirus*). The autophagy protein ATG6 serves as a receptor for the NIb RNA-dependent RNA polymerase of *Turnip mosaic virus* (TuMV, *Potyvirus*). The selective autophagy of the HCPro silencing suppressor of *Tobacco etch virus* (TEV) requires calmodulin-like protein rgs-CaM as an intermediary. The rgs-CaM binding partner on the phagophore is unknown. Right panel: Two examples of proviral functions of selective autophagy. During TuMV infection, the NIb viral RNA polymerase interacts with the NBR1 autophagy receptor and ATG8f, and associates with TONOPLAST INTRINSIC PROTEIN 1 (TIP1) and viral 6K2 proteins. This complex triggers a formation of tonoplast-bound replication compartments, protected from plant defenses. The P0 viral protein of *Turnip yellows virus* (TuYV, *Polerovirus*) forms a complex with ubiquitinated ARGONAUTE1, a key component of RNA interference pathway, and ATG8-INTERACTING PROTEIN1 (ATI1), which is subsequently degraded by ATG5/ATG7-mediated autophagy, resulting in a decreased RNA interference activity.

In conclusion, autophagy may play both antiviral and proviral roles during viral replication in plant cells (**Figure 3**). Regarding the regulation of the autophagy processes, recent findings indicate that autophagy activation is tightly linked to the unfolded protein response, which is triggered by various plant viruses. In a process termed IRE1-dependent decay of messenger RNA (RIDD), IRE1b degrades the RNA transcripts of factors that interfere with the induction of autophagy in Arabidopsis (Liu et al., 2012; Yang et al., 2016). In addition, the activation of UPR is directly linked to upregulation of genes encoding selective autophagy receptors (Li et al., 2020a). However, a limited information is available on the contribution of the two major autophagy regulatory hubs, represented by the TOR complexes and kinases from the SnRK1 family, on plant–virus interactions. The current available data indicate that TOR inhibition causes increased resistance to *Watermelon mosaic virus* (WMV, +ssRNA, genus *Potyvirus*) and *Tobacco mosaic virus* (*Tobamovirus*) (Ouibrahim et al., 2015; Marash et al., 2022).

## 5 Concluding remarks

Plant cells contain a diverse population of membrane structures that perform distinct tasks in plant development and in responses to environmental cues. Understanding the molecular mechanisms of the host endomembrane system misappropriation by positive-strand RNA viruses provides invaluable insights into processes of viral replication, assembly, and intercellular movement. Recent reports on the participation of membrane-localized receptor-like kinases in detection of viral nucleic acids indicate that the classical pattern-triggered immunity, crucial for detection of bacterial and fungal pathogens, comprises an important part of antiviral defenses in plant cells. The emerging knowledge of plant selective autophagy indicates its dual role in plant–virus interactions. Selective autophagy targets viral components for degradation to restrict viral accumulation, while being manipulated by viral proteins to promote viral infection. The detailed comprehension of molecular mechanisms discussed partly in this review will likely lead to the development of potential biotechnology strategies to enhance and engineer crop resistance to viral infections.

## Author contributions

IJ, NF and JZ wrote sections of the manuscript and prepared the figures. All authors contributed to the article and approved the submitted version.
